# Supplementary material for: The increasing burden of group B Streptococcus from 2013 to 2023: a retrospective cohort study in Beijing, China
Source: Microbiol Spectr. 2024 Dec 10;13(1):e02266-24. doi: 10.1128/spectrum.02266-24 (PMC11705810; doi:10.1128/spectrum.02266-24)
Supplement: Figure S1 — Total number of swabs that tested positive for pathogens and proportion of GBS in vaginal pathogens for each group per year. [file spectrum.02266-24-s0001.docx]

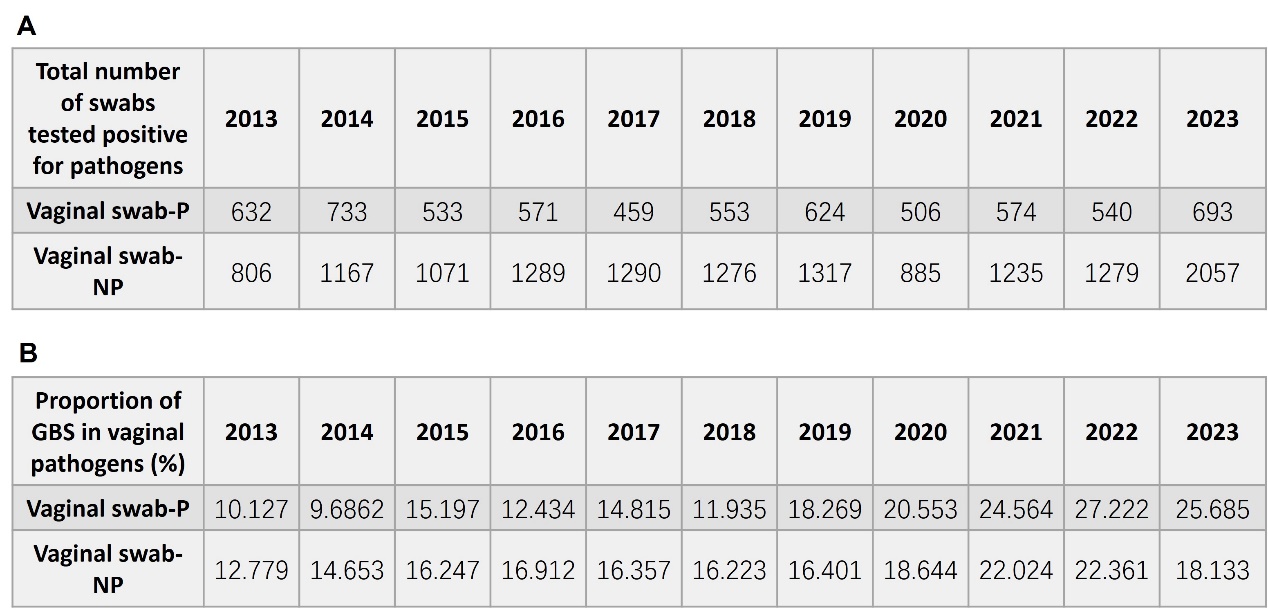


**Figure S1** The total number of swabs tested positive for pathogens (A) and proportion of GBS in vaginal pathogens (%) (B) for each group per year.
